# Health-related quality of life (HRQoL) loss associated with self-perceived anxiety/depression in seropositive rheumatoid arthritis

**DOI:** 10.1007/s10067-024-07186-x

**Published:** 2024-10-30

**Authors:** Diego Fernando Rojas-Gualdrón, Carolina Franco-Salazar, Clara Ángela Gómez-Henck, Maria Camila Manrique-Castrillón, Yennifer Carime Hoyos-Méndez, Susana Vélez-Romero, Juan Camilo Díaz-Coronado

**Affiliations:** 1 Facultad de Medicina, Universidad CES, Calle 10ª # 22-04 Bloque C Piso 2, Medellín, Colombia; 2 ARTMEDICA IPS, Medellín, Colombia

**Keywords:** Anxiety, Arthritis, Depression, Quality-adjusted life years, Rheumatoid

## Abstract

**Objective:**

To analyze the HRQoL loss associated with self-perceived anxiety/depression in patients with seropositive rheumatoid arthritis (RA).

**Method:**

This secondary data analysis is based on a registry-based retrospective follow-up study of patients with seropositive RA treated between August 2014 and January 2023 in ARTMEDICA, Colombia. HRQoL loss and self-perceived anxiety/depression were defined as outcomes. Disease activity (DAS-28) and other patient data were also gathered. Statistical analyses were performed using the ordinal logistic and generalized linear regression models.

**Results:**

A total of 3579 patients with a mean follow-up of 2.9 (SD 2.4) years, 85.6% women with a median age at diagnosis of 48.1 (IQR 37.8–57.5) years, and a median of 6.5 (IQR 1.9–14.7) years living with RA were included. At program admission, the median DAS-28 score was 2.8 (IQR 2.1–4.2), and 6.6% of patients reported extreme anxiety/depression. The average HRQoL loss was 3.4 months per year lived with seropositive AR. Among patients with no pain or discomfort, moderate and extreme anxiety/depression were associated with mean HRQoL losses of 2.2 (95% CI − 2.3 to − 2.2) and 4.1 (95% CI − 4.3 to − 3.8) months. In patients with extreme pain/discomfort, these estimations were 0.8 (95% CI − 0.9 to − 0.7) and 1.9 (95% CI − 2.1 to − 1.7) months, respectively.

**Conclusion:**

Our study adds to the available body of evidence by clarifying the differential impact of anxiety/depression on HRQoL, depending on the severity of pain. These findings highlight the importance of strengthening mental health care and psychological well-being interventions for patients with RA, regardless of pain or disease activity.

**Table:** 

**Key Points** • *The average HRQoL loss was 3.4 months per year lived with seropositive AR.* • *Pain/discomfort rather than disease activity explained the severity of anxiety/depression as well as its associated HRQoL loss.* • *For patients with extreme pain/discomfort and anxiety/depression, the average HRQoL loss was 8.1 months per year lived with the disease compared to 0.4 months for patients without those impacts.*

## Introduction

Rheumatoid arthritis (RA) is a chronic and progressive autoimmune disease that affects an estimated 16 to 20 million people worldwide [1, 2]. Fluctuation in the disease over time impacts not only physical but also psychological functioning [3], as chronic pain and disease activity have been associated with an increased risk of depression and anxiety [4]. Anxiety and depression are frequent in RA, with prevalences commonly reported in the range of 14 to 40% [5].

Although previous studies have shown how mental health issues are associated with worse outcomes, in terms of disease activity, during treatment of patients with early RA [6], intervention studies of depression and anxiety for patients with RA are scarce [7]. Proper identification and management of mental health issues in people living with RA may contribute to improved outcomes, particularly patient-centered outcomes such as health-related quality of life (HRQoL).

Understanding how much HRQoL loss is associated with mental health provides evidence to support the need to recognize and appropriately treat this significantly affected aspect in patients with RA. However, evidence, particularly from Latin American populations, is scarce. So, this study aimed to analyze the HRQoL loss associated with self-perceived anxiety/depression in patients with seropositive RA from a large cohort of Colombian patients enrolled in the ARTMEDICA surveillance and follow-up program for autoimmune diseases.

## Methods

The original study on which this secondary analysis was nested was approved by the CES University Institutional Review Board and was executed following the Council for International Organizations of Medical Sciences-CIOMS ethical guidelines for registry-based health research. Upon admission to the program, informed consent was obtained from patients for using their medical history data under conditions of confidentiality and anonymity for research purposes. This report follows the recommendations of the STROBE statement [8].

### Study design and setting

A registry-based retrospective follow-up study was conducted from admission to the ARTMEDICA surveillance and follow-up program for autoimmune diseases until the last recorded medical assessment. This program comprises a qualified team of rheumatologists, general practitioners, pharmacists, nurses, and psychologists, providing a comprehensive risk management protocol to positively impact morbidity, mortality, functionality, and costs.

The study included registries of patients treated between August 2014 and January 2023. The data was extracted in March 2023.

### Participants

We included all patients who met the following criteria: (1) diagnosed with seropositive RA, (2) enrolled in the ARTMEDICA program, (3) with a baseline measurement of HRQoL within the first month, and (4) with at least one follow-up measure of HRQoL. Patients without concurrent measures of disease activity (DAS-28) were excluded. Patient selection and follow-up were based on linked administrative and clinical records.

### Variables and measurement

The primary outcome was the HRQoL loss measured with the EQ-5D-3L health state utility (HSU) [9]. It quantifies the number of months lived with optimal QoL lost per year lived with the disease. Following the instructions of the Colombian Institute for Health Technology Assessment, the Hispanic valuation of the EQ-5D health states was used [10]. Self-perceived anxiety/depression measured with the fifth dimension of the EQ-5D-3L instrument was defined as the secondary outcome variable. This dimension has three levels of response: (1) “I am not anxious or depressed,” (2) “I am moderately anxious or depressed,” and (3) “I am extremely anxious or depressed.” The EQ-5D-3L is a valuable screening tool with an area under the ROC curve of 0.86 to detect symptoms of anxiety and depression and 0.91 to detect moderate-severe symptoms [11].

As potential explanatory factors, patient self-perceived impact on the other four dimensions of the EQ-5D-3L: mobility, self-care, usual activities, pain/discomfort, and repeated measures of disease activity with the DAS-28 score, among other clinical and demographic characteristics, were also included. Cut-off values for classifying disease activity were as follows: remission (< 2.6), low (≤ 3.2), moderate (≤ 5.1), and high activity (> 5.1) [12].

### Bias

We collected data from structured clinical records filled out by the attending rheumatologists, and the selection of patients was based on the ICD-10 code M05.9. During the study period, the institution adopted the Colombian clinical practice guideline recommendations for early detection, diagnosis, and treatment of AR, which recommends the 2010 classification criteria from the American College of Rheumatology-ACR and the European League Against Rheumatism (EULAR). The linkage of administrative and clinical records was based on unique patient registration codes.

### Study size

There was no a priori estimation of sample size. Post hoc analysis established that the studied sample size (*n* = 3579) had a precision of ± 0.2 months for mean differences in HRQoL loss, assuming 95% confidence and 95% coverage probability.

### Statistical methods

Categorical variables were analyzed using percentages, and quantitative variables using median and interquartile range (IQR). The analyses of HRQoL loss were performed using the generalized linear model (GLM) with cluster estimation of variance (measures clustered within patients). Results are presented as mean and mean differences (mean diff.) with 95% confidence intervals (95% CI) and *p*-values. The analyses of the severity of anxiety/depression were performed using an ordinal logistic regression with cluster estimation of variance. Results are presented as percentages and ordinal Odds Ratio (oOR) with 95% CI and *p*-values. Interactions between disease activity and pain/discomfort for explaining anxiety/depression and between anxiety/depression and pain/discomfort for explaining HRQoL loss were explored; all results related to interactions are presented as marginal estimates. All estimates were adjusted for the potential confounding effect of differences in sex, age at diagnosis, age at program admission, time lived with the disease, self-perceived health, and impact on EQ-5D-3L dimensions.

Sensitivity analyses were performed with different distribution families for GLM and probit for ordinal regression. There was no missing data; all analyses were performed on the full dataset. Statistical analyses were obtained in Stata version 16.1 (College Station, TX).

## Results

### Participants

The original study included 11,099 patients with seropositive RA; 1518 patients were not eligible as they had no baseline (within the first month), and 5398 patients were not included as they had not at least one follow-up measure of the EQ-5D-3L. The remaining 3880 (35.0%) patients fulfilled the inclusion criteria, among whom 301 (7.8%) were excluded as they did not have concurrent disease activity and EQ-5D-3L measures. Finally, 3579 patients contributing 10,342 years (mean follow-up 2.9 years, SD 2.4) were included and analyzed.

There were no relevant differences among included and not included patients at baseline in median disease activity (2.8 vs. 2.6), age at diagnosis (48.1 vs. 49.3), or years lived with the disease (6.5 vs. 6.0). The only relevant difference was in the years spent in the program (2.5 vs. 0.5).

### Descriptive data

At program admission (Table 1), the median time lived with seropositive AR was 6.5 years (IQR 1.9–14.7) with a median HSU of 0.69 (IQR 0.60–0.77). The median DAS-28 score was 2.8 (IQR 2.1–4.2), with 28.1% and 13.3% of patients in moderate and high activity, respectively. The median age at AR diagnosis was 48.1 years (IQR 37.8–57.5), and 85.6% of patients were women. Concerning HRQoL, pain/discomfort was the most affected dimension, with 18.3% of patients reporting extreme pain or discomfort, followed by anxiety/depression, with 6.6% of patients feeling extremely anxious or depressed.

**Table 1:** Patient characteristics and health-related quality of life (HRQoL) at program admission

	***n***	**%**
Sex		
Woman	3064	85.6
Man	515	14.4
Years of schooling, median (IQR)	8	(5–11)
Age at diagnosis, median (IQR)	48.1	(37.8–57.5)
Years lived with the disease, median (IQR)	6.5	(1.9–14.7)
Self-perceived health, median (IQR)	0.70	(0.50–0.80)
Health state utility, median (IQR)	0.69	(0.60–0.77)
Disease activity (DAS-28)		
Median (IQR)	2.8	(2.1–4.2)
Remission (< 2.6)	1637	45.7
Low (≤ 3.2)	459	12.8
Moderate (≤ 5.1)	1007	28.1
High (> 5.1)	476	13.3
EQ-5D-3L dimensions		
Mobility		
I have no problems in walking about	1830	51.1
I have some problems in walking about	1702	47.6
I am confined to bed	47	1.3
Self-care		
I have no problems with self-care	2332	65.2
I have some problems washing or dressing myself	1217	34.0
I am unable to wash or dress myself	30	0.8
Usual activities		
I have no problems with performing my usual activities	1910	53.4
I have some problems with performing my usual activities	1573	44.0
I am unable to perform my usual activities	96	2.7
Pain/discomfort		
I have no pain or discomfort	975	27.2
I have moderate pain or discomfort	1948	54.4
I have extreme pain or discomfort	656	18.3
Anxiety/depression		
I am not anxious or depressed	2353	65.7
I am moderately anxious or depressed	990	27.7
I am extremely anxious or depressed	236	6.6

*IQR* interquartile range

### Outcome data

**Fig. 1:**
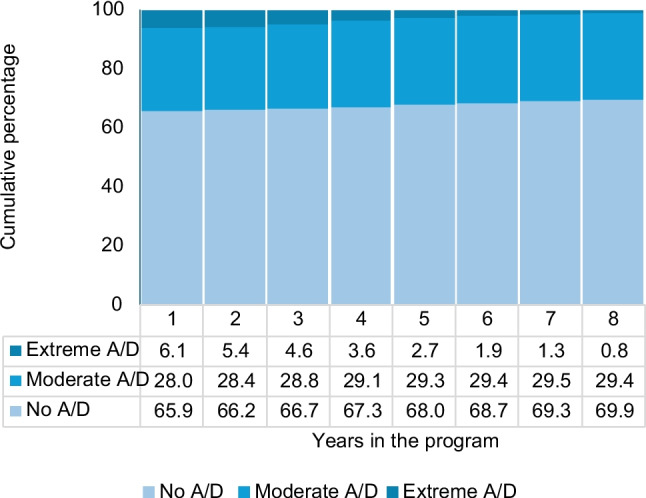
Severity of self-reported anxiety/depression by years in the program. This figure shows how the prevalence of extreme anxiety/depression decreases as patients spend more years in the ARTMEDICA surveillance and follow-up program for autoimmune diseases. However, the prevalence of moderate anxiety depression remains relatively steady. *A/D* anxiety/depression. Adjusted by the variables presented in Table 2

Of the analyzed 10,342 patient-years, patients spent an equivalent of 7414.7 years with optimal QoL and experienced a HRQoL loss equivalent to 2927.3 years, for an average HRQoL loss of 3.4 months per year lived with seropositive AR. Concerning the severity of anxiety/depression, it presented a statistically significant trend of reduction over time with an oOR of 0.98 (95% CI 0.96–0.99) per each additional year in the program (Table 2). After 1 year in the program, the percentage of severely affected patients was 6.1%, a figure that reduced to 2.7% at year five and 0.8% at year eight (Fig. 1). However, moderate anxiety/depression remained relatively steady between 28 and 29.4% in years one and eight, respectively.

**Table 2:** Factors associated with the severity of self-reported anxiety/depression

	oOR	*p*-value	95% CI	
Years in the program, per additional year	0.98	0.008	(0.96–0.99)	
Disease activity (DAS-28)				
Remission (< 2.6)	1.00			
Low (≤ 3.2)	1.03	0.517	(0.93–1.15)	
Moderate (≤ 5.1)	1.07	0.079	(0.99–1.17)	
High (> 5.1)	1.11	0.034	(1.01–1.23)	
Sex				
Woman	1.43	< 0.001	(1.29–1.59)	
Man	1.00			
Years of schooling, per additional year	0.99	0.023	(0.98–1.01)	
Age at diagnosis, per additional year	0.99	< 0.001	(0.99–0.99)	
Self-perceived health, per additional 10%	0.92	< 0.001	(0.91–0.93)	
EQ-5D-3L dimensions				
Mobility				
I have no problems in walking about	1.00			
I have some problems in walking about	1.69	< 0.001	(1.56–1.84)	
I am confined to bed	1.93	0.001	(1.32–2.83)	
Self-care				
I have no problems with self-care	1.00			
I have some problems washing or dressing myself	2.03	< 0.001	(1.85–2.24)	
I am unable to wash or dress myself	2.26	0.001	(1.40–3.64)	
Usual activities				
I have no problems with performing my usual activities	1.00			
I have some problems with performing my usual activities	1.19	< 0.001	(1.08–1.31)	
I am unable to perform my usual activities	1.84	< 0.001	(1.38–2.46)	
Pain/discomfort				
I have no pain or discomfort	1.00			
I have moderate pain or discomfort	2.55	< 0.001	(2.30–2.83)	
I have extreme pain or discomfort	4.88	< 0.001	(4.27–5.59)	

*oOR* ordinal Odds Ratio, *95% CI* 95% confidence interval

### Main results

**Fig. 2:**
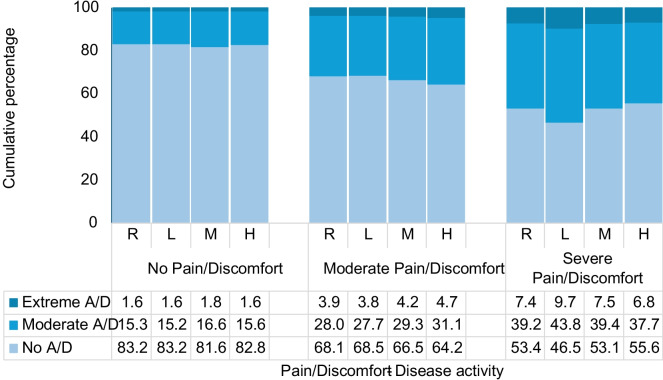
Severity of self-reported anxiety/depression according to disease activity and self-reported pain/discomfort. The figure shows how the severity of anxiety/depression is mainly explained by pain/discomfort, not by disease activity (DAS-28). *A/D* anxiety/depression. Disease activity (DAS-28): *R* remission (< 2.6), *L* low (≤ 3.2), *M* moderate (≤ 5.1), *H* high (> 5.1). Marginal estimates were obtained at 1 year of follow-up and were adjusted by the variables presented in Table 2

Extreme pain/discomfort (oOR 4.88; 95% CI 4.27–5.59) and high disease activity (oOR 1.11; 95% CI 1.01–1.23) were the most relevant HRQoL-related and clinical characteristics among those significantly associated with the severity of anxiety/depression (Table 2). However, the analysis of their interaction revealed that pain/discomfort is the one that explains anxiety/depression. As presented in Fig. 2, the severity of anxiety/depression differs between groups of pain/discomfort but not between groups of disease activity.

**Fig. 3:**
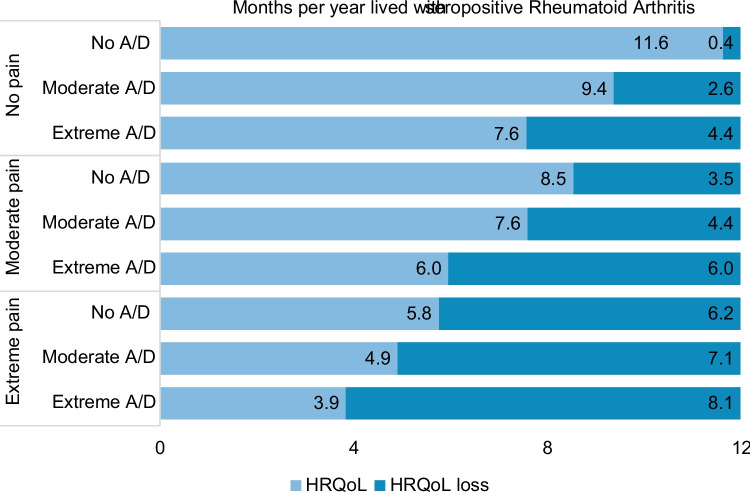
Health-related quality of life (HRQoL) loss per year lived with the disease according to the severity of self-perceived anxiety/depression and pain/discomfort. This figure presents how the HRQoL loss associated with self-perceived anxiety/depression is higher among patients without self-perceived pain/discomfort but still relevant among patients with moderate and severe pain/discomfort. *A/D* anxiety/depression, *HRQoL* health-related quality of life. Marginal estimates were adjusted by the variables presented in Table 2

Patients without pain/discomfort or anxiety/depression experienced a HRQoL loss of 0.4 months per year lived with seropositive AR. For patients only experiencing extreme anxiety/depression, the mean loss was 4.4 months, and for patients only experiencing extreme pain/discomfort, the mean loss was 6.2 months. However, for patients experiencing extreme affectation in both dimensions, the mean HRQoL loss was 8.1 months (Fig. 3). The HRQoL loss was statistically significant for all levels of self-perceived anxiety/depression (Table 3). However, it differed depending on pain/discomfort in the range of 0.8 (95% CI − 0.9 to − 0.7) additional months of HRQoL loss for moderate anxiety/depression among patients with extreme pain/discomfort to 4.1 (95% CI − 4.3 to − 3.8) additional months of HRQoL loss for extreme anxiety/depression among patients with no pain/discomfort.

**Table 3:** Health-related quality of life (HRQoL) loss associated with self-perceived anxiety/depression according to self-perceived pain/discomfort

Pain/discomfort	Anxiety/depression	Mean diff	95% CI
No pain/discomfort	No A/D	0.0	
	Moderate A/D	− 2.2	(− 2.3 to − 2.2)
	Extreme A/D	− 4.1	(− 4.3 to − 3.8)
Moderate pain/discomfort	No A/D	0.0	
	Moderate A/D	− 0.9	(− 1.0 to − 0.9)
	Extreme A/D	− 2.6	(− 2.7 to − 2.5)
Extreme pain/discomfort	No A/D	0.0	
	Moderate A/D	− 0.8	(− 0.9 to − 0.7)
	Extreme A/D	− 1.9	(− 2.1 to − 1.7)

*Mean diff.* mean difference, in months; *95% CI* 95% confidence interval; *A/D* anxiety/depression. Mean differences were adjusted by the variables presented in Table 2

### Sensitivity analysis

GLM estimates of mean HRQoL loss with Gaussian distribution were similar to those obtained with robust Poisson. However, estimations with a Gamma distribution produced smaller but statistically significant differences in HRQoL by anxiety/depression: − 0.9 (95% CI − 1.5 to − 0.4), − 2.1 (95% CI − 2.8 to − 1.5), and − 2.8 (95% CI − 2.0 to − 0.4) months per year lived with the disease for patients with extreme, moderate, and without pain/discomfort, respectively. On the other hand, sensitivity analysis of factors associated with the severity of anxiety/depression produced similar results with the ordinal logit and ordinal probit models.

## Discussion

This study aimed to analyze the HRQoL loss associated with self-perceived anxiety/depression in patients with seropositive RA, finding that anxiety/depression produces a loss equivalent to 0.8 to 4.1 additional months of HRQoL per year lived with the disease, depending on the severity of pain/discomfort. Remarkably, among patients with concurrent severe anxiety/depression and severe pain/discomfort, the loss is equivalent to 8.1 months per year.

Self-perceived pain/discomfort rather than disease activity was the most relevant contributor to the severity of anxiety/depression and its related HRQoL loss. This finding is consistent with previous studies highlighting the role of pain, particularly residual and non-inflammatory pain, on the impact of RA on quality of life and psychological well-being [13]. In the study by Ziarko et al. (2019), pain intensity was associated with anxiety and depression in RA patients with and without biological treatment [14]. Panjrattan et al. (2023) reported that clinical disease activity and pain index were independently associated with depression [15]; however, the authors did not perform a statistical analysis that considered the high correlation between pain and disease activity.

Regarding the effect of depression and anxiety on HRQoL, Beşirli et al. (2020) reported that the presence of concomitant anxiety and depression in patients with RA was associated with suicidal ideation and poor quality of life [16]. Ozcetin et al. (2007) reported that the strong negative correlations observed in patients with RA were similar to those observed in patients with knee osteoarthritis and fibromyalgia syndrome [17]. The negative association between anxiety and HRQoL in patients with RA was confirmed by a meta-analysis by Machin et al. (2020), who reported reduced physical (*r* =  − 0.39, CI − 0.57, − 0.20) and mental QoL (− 0.50, CI − 0.57, − 0.43) [5]. However, as far as the authors are aware, this is the first study to determine the HRQoL loss associated with anxiety/depression in seropositive AR patients. Despite known clinical and treatment response differences, no studies compare HRQoL loss between seropositive and seronegative AR [18]. Understanding the differential mental health impact of seropositivity in patients with AR may provide valuable insights to improve their HRQoL.

About the differential HRQoL loss associated with anxiety/depression, depending on pain/discomfort, from the perspective of the biopsychosocial model of pain, evidence suggests that both cognitive and emotional processes contribute to interindividual differences in perception and impact of pain, potentially associating pain catastrophizing with pain severity, sensitivity, disability, and poor treatment outcomes [19]. In this sense, studies suggest that initiating adequate and timely patient education focusing on positive communication and self-efficacy [19] and early mental illness detection and treatment [20] may positively impact HRQoL in RA. However, further studies aiming for an in-depth characterization of pain and pain perception and management-related processes are required to fully understand its interaction with anxiety and depression to improve HRQoL.

Psychological interventions for patients with RA have evidence of small to moderate improvements in anxiety, depression, pain, and HRQoL. Cognitive-behavioral and alternative therapies such as meditation, mindfulness, or yoga have shown positive benefits in psychosocial outcomes related to disease activity, particularly among RA patients with comorbid depression [21, 22]. However, according to Stoll et al. (2024), more than adding psychological interventions to conventional care, a holistic approach considering biopsychosocial factors is required to fully consider complex interactions between the disease, mental health, and QoL in patients living with RA [23]. The evidence on pharmacological interventions for anxiety and depression in patients with RA is limited [7].

In our study, severe anxiety/depression tended to decrease as patients spent more time in the surveillance and follow-up program; however, moderate anxiety depression remained relatively steady. This level of affectation was associated with 1 to 2 additional months of HRQoL loss per year lived with seropositive RA. Moderate symptoms of anxiety or depression may go unnoticed but still may influence the effectiveness of treatment and the prognosis of the disease [24], leading to increased use of health services, healthcare costs, and reduced work productivity [25]. The need for multidisciplinary care that encompasses the mental health component has already been recognized in clinical practice guidelines for other chronic pain conditions such as fibromyalgia [26], migraine [27], and chronic low back pain [28].

This study has limitations. As a registry-based study, it has the usual limitations of a retrospective study regarding data quality and availability. Patient selection was based on registered ICD-10 codes, and even when all rheumatologists follow the same institutionally adopted clinical practice recommendations for early detection and diagnosis, potential variations in coding practices between professionals and inter-rater reliability are unknown. Residual confounding is also a limitation of this study. We adjusted our estimates of HRQoL loss by disease activity, time lived with the disease, other dimensions of QoL, age, and sex. However, other clinical, patient, and treatment-related characteristics relevant to pain, such as comorbidities, non-inflammatory pain mechanisms, the role of central sensitization, or structural joint damage, were not adjusted. Further studies should try to understand if those characteristics may explain the HRQoL loss associated with anxiety/depression.

An additional relevant limitation is that all patients come from the same follow-up program and are not a statistically representative sample of all Colombian patients. However, ARTMEDICA has care centers in nine out of 32 administrative departments in Colombia, which capture relevant social, economic, and geographical heterogeneity. Additionally, we excluded 7.8% of eligible patients due to a lack of concurrent data on disease activity and HSU, which also may limit the generalizability of the results.

In terms of potential information bias, disease activity was measured with the DAS-28, which, despite some comparative limitations with other measures such as the Clinical Disease Activity Index (CDAI) or the Simplified Disease Activity Index (SDAI), is still one of the five measures recommended for use in regular clinical settings [29]. Additionally, we measured HRQoL loss and self-perceived severity of anxiety/depression with the EQ-5D-3L, which is highly comparable and allows the estimation of QALYs but does not capture specific relevant health impacts for patients with AR, considered in specific HRQoL instruments such as the Patient Reported Outcome Measurement Scale Physical Function (PROMIS-PF) included in the RA measures toolkit of the American College of Rheumatology [30].

Despite limitations, our results provide a general portrait of the interaction between anxiety/depression and pain/discomfort on the HRQoL loss. Further studies are required to identify explanatory mechanisms that provide insight into the optimal mental health interventions for these patients.

## Conclusion

The joint presentation of moderate to severe pain/discomfort and anxiety/depression produces a significant HRQoL burden for patients living with seropositive RA. Even in patients who are not experiencing pain/discomfort, the mental health impact of the disease produces a relevant loss of HRQoL. Our study adds to the available body of evidence by clarifying the differential impact of anxiety/depression on HRQoL, depending on the severity of pain/discomfort. These findings highlight the importance of strengthening mental health care and psychological well-being interventions for patients with RA, regardless of pain or disease activity.

## Data Availability

The data supporting this study’s findings are available from the corresponding author upon reasonable request.
